# Zn_2_SnO_4_@SiO_2_@5-FU Nanoparticles as an Additive for Maxillary Bone Defects

**DOI:** 10.3390/ijms26010194

**Published:** 2024-12-29

**Authors:** Ana Maria Gianina Rehner (Costache), Andreea Gabriela Bratu, Alexandra Cătălina Bîrcă, Adelina-Gabriela Niculescu, Alina Maria Holban, Ariana Hudiță, Florentina Cornelia Bîclesanu, Paul Cătălin Balaure, Anna Maria Pangică, Alexandru Mihai Grumezescu, George-Alexandru Croitoru

**Affiliations:** 1 Faculty of Medicine, Titu Maiorescu University, 031593 Bucharest, Romania; rehner.ana@gmail.com (A.M.G.R.); corneliabicle@yahoo.com (F.C.B.); anna-maria.pangica@prof.utm.ro (A.M.P.); 2 Faculty of Chemical Engineering and Biotechnologies, University Politehnica of Bucharest, Gh. Polizu St. 1-7, 060042 Bucharest, Romania; gabriela.bratu@stud.fim.upb.ro (A.G.B.); ada_birca@yahoo.com (A.C.B.); adelina.niculescu@upb.ro (A.-G.N.); paul.balaure@upb.ro (P.C.B.); 3 Research Institute of the University of Bucharest—ICUB, University of Bucharest, 050657 Bucharest, Romania; arianahudita@yahoo.com; 4 Faculty of Biology, University of Bucharest, Aleea Portocalelor 1-3, Sector 5, 77206 Bucharest, Romania; alina_m_h@yahoo.com; 5 Faculty of Dental Medicine, Carol Davila University of Medicine and Pharmacy, 8 Eroii Sanitari Street, 050474 Bucharest, Romania; alex.croitoru@umfcd.ro

**Keywords:** Zn_2_SnO_4_ nanoparticles, SiO_2_ coating, 5-Fluorouracil, antibacterial activity, antitumor therapy, nanoparticle stability, biofilm inhibition, A-431 cell line, drug delivery, biomedical applications

## Abstract

This study investigates the synthesis of Zn_2_SnO_4_@SiO_2_@5-FU nanoparticles as an additive for bone fillers in dental maxillofacial reconstruction. Zn_2_SnO_4_ nanoparticles were synthesized and coated with a SiO_2_ shell, followed by the incorporation of 5-Fluorouracil (5-FU), aimed at enhancing the therapeutic properties of classical fillers. Structural analysis using X-ray diffraction confirmed that Zn_2_SnO_4_ was the single crystalline phase present, with its crystallinity preserved after both SiO_2_ coating and 5-FU incorporation. SEM characterization revealed the micro-spherical particles of Zn_2_SnO_4_ assembled by an agglomeration of nanorods, exhibiting dimensions and morphological characteristics that were consistent after the addition of both the SiO_2_ shell and 5-FU. Fourier-transformed infrared spectroscopy provided solid proof of the successful synthesis of Zn_2_SnO_4_, Zn_2_SnO_4_@SiO_2_, and Zn_2_SnO_4_@SiO_2_@5-FU, confirming the presence of expected functional groups. The SiO_2_ layer improved nanoparticle stability in the solution, as indicated by zeta potential measurements, while adding 5-FU significantly increased biocompatibility and targeting efficiency. The existence of the SiO_2_ shell and 5-FU is also confirmed by the hydrodynamic diameter, indicating an increase in particle size after incorporating both compounds. Antibacterial assays demonstrated a selective efficacy against Gram-positive bacteria, with Zn_2_SnO_4_@SiO_2_@5-FU showing the strongest inhibitory effects. Biofilm inhibition studies further confirmed the nanoparticles’ effectiveness in preventing bacterial colonization. Cytotoxicity tests on the A-431 human epidermoid carcinoma cell line revealed a dose-dependent reduction in cell viability, highlighting the potential of 5-FU for targeted cancer treatment. These findings highlight the potential of Zn_2_SnO_4_@SiO_2_@5-FU nanoparticles as a multifunctional additive for bone fillers, offering enhanced antimicrobial and antitumor capabilities.

## 1. Introduction

Bone fillers are critical in dental maxillofacial reconstruction, aiding in restoring and integrating bone structures that have been compromised due to injury, disease, or surgical procedures [1]. Dental maxillofacial bone repair involves irregular anatomy, equilibrium between hosts and oral cavity microbes, and advanced periodontal structures that promote epithelial growth. Consequently, oral maxillofacial reconstruction needs replacement materials that meet rigorous and precise standards [2,3,4]. Biocompatible materials are crucial in conventional treatments because of the specific requirements for advancements in clinical therapy and tissue regeneration [5,6]. Despite the advancements in biomaterials, traditional bone fillers face challenges such as the risk of infection, inadequate integration with host tissue, and limited stability. Unfortunately, the conventional clinical therapies for maxillofacial tumors (radiotherapy, chemotherapy, and surgery) are harmful to the host, leading to insufficient therapeutic results such as physiological limitations, including difficulties with speech, swallowing, chewing, sucking, and breathing, as well as tumor recurrence. At the same time, dental materials may stimulate immunological responses, resulting in inflammation or rejection, leading to tissue fibrosis growth and the resorption of the alveolar bone [7,8]. There is a growing need for bone fillers that support bone regeneration and exhibit antimicrobial and antitumor properties to enhance treatment outcomes [9].

Nanotechnology has emerged as a promising approach in dental and medical applications, offering unique advantages in terms of size, structure, surface area, and bioactivity [10]. Bone substitutes are classified as bone grafts (autograft, allograft, and xenograft), ceramics/synthetics (hydroxyapatite and tricalcium phosphate), and growth factors (human demineralized bone matrix) [11,12,13]. Autologous bone grafting was once considered the gold standard for bone filling, providing all characteristics for bone regeneration: osteoinductivity, osteoconductivity, and osteogenicity. However, the limitations of donors have restricted its development. Exogenous bone replacement materials are susceptible to immune reactions and disease transmission, and artificial material grafts make it challenging to achieve optimal therapeutic results due to their lack of osteogenic induction characteristics [8,14,15]. Incorporating nanomaterials into bone fillers can significantly improve their mechanical properties, enhance bone regeneration, support and regulate cellular function, proliferation, and migration, and provide effective antimicrobial activity. These benefits make nanoparticles ideal candidates for advanced biomedical applications, particularly in enhancing bone filler performance in dental maxillofacial reconstruction [16,17,18,19].

Zn_2_SnO_4_ belongs to the class of ternary oxides, which possess remarkable characteristics and can be synthesized in different morphologies. These nanoparticles have garnered attention due to their distinctive properties, including high crystallinity, non-toxicity, chemical stability, antioxidant properties, pH-dependent properties, optical properties, sustained release of zinc ions, and potential antimicrobial capabilities [20,21,22,23,24]. Specifically, Zn_2_SnO_4_ nanoparticles exhibit a higher availability of reactive oxygen species (ROS) than similar nanosystems (i.e., ZnO and SnO_2_ nanoparticles), leading to enhanced antibacterial activity. Moreover, Zn^2+^/Sn^4+^ ions released from Zn_2_SnO_4_ are attracted to the negatively charged bacterial cell membrane. These ions penetrate the membrane, interact with sulfhydryl groups in membrane proteins, and disrupt enzymatic activity, including synthetase function. This interference hampers cell division, ultimately causing bacterial cell death [20,25]. These characteristics make Zn_2_SnO_4_ an attractive material for biomedical applications displaying excellent antibacterial properties toward relevant pathogens [22,26,27,28]. Thus, these nanomaterials can be of interest for enhancing bone fillers’ structural and antimicrobial properties by interacting with microbial cell membranes.

Furthermore, zinc stannate can display an antitumoral effect by raising the levels of ROS inside the malignant cell, and, eventually, oxidative stress damages the constituents of the cell. Antitumor effects have been noted to be dose-dependent, with higher Zn_2_SnO_4_ nanoparticle concentrations providing elevated intracellular ROS levels in exposed cancer cells. The oxidative damage has been further linked with a reduction in cancer cell migration and proliferation, with a significant decrease in metastasis risks [20,21,22,23,24]. Moreover, Zn_2_SnO_4_ nanoparticles can be functionalized for improved tumor cell targeting and internalization [29], holding promise as theranostic tools. In this context, the development of Zn_2_SnO_4_-based nanomaterials could address the limitations of conventional bone fillers, providing a multifunctional solution for complex dental reconstructions.

To further improve the performance of Zn_2_SnO_4_ nanoparticles, surface modifications were employed using a SiO_2_ shell and 5-Fluorouracil (5-FU). The SiO_2_ coating enhances the stability and dispersibility of the nanoparticles in solution [30,31], while 5-FU, a known chemotherapeutic and antibacterial agent, introduces supplementary antitumor and antimicrobial properties [32,33,34,35]. This dual modification aims to create a multifunctional nanoparticle that can facilitate bone regeneration, prevent infections, and potentially target tumor cells, making it suitable for use in dental maxillofacial applications.

The primary objective of this study is to develop multifunctional Zn_2_SnO_4_@SiO_2_@5-FU nanoparticles as an additive for bone fillers in dental maxillofacial reconstruction. This work aims to evaluate the structural integrity, biocompatibility, antimicrobial efficacy, and antitumor potential of these modified nanoparticles. By addressing the limitations of existing bone fillers, this research seeks to contribute to developing more effective materials for reconstructive dental applications.

## 2. Results

### 2.1. X-Ray Diffraction Spectrum (XRD)

**Figure 1:**
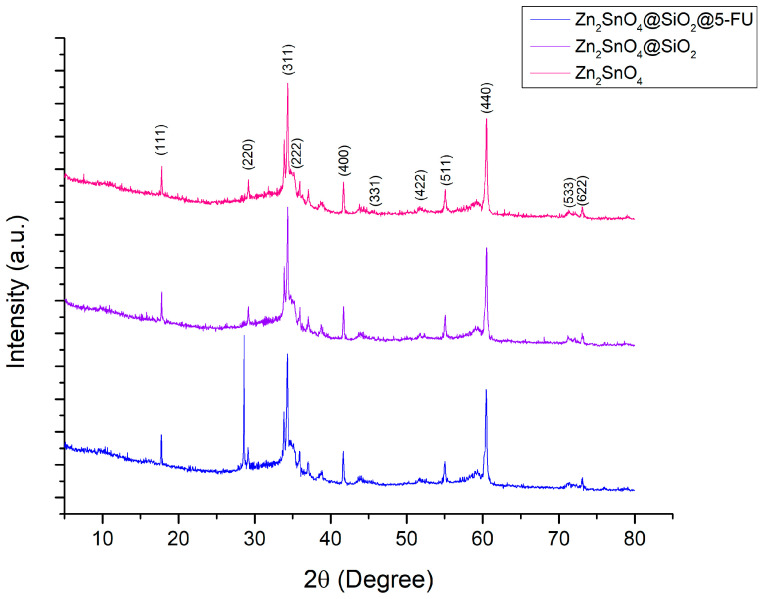
XRD patterns of Zn_2_SnO_4_, Zn_2_SnO_4_@SiO_2_, and Zn_2_SnO_4_@SiO_2_@5-FU samples.

The phase purity and crystallinity of Zn_2_SnO_4_ nanoparticles were characterized using X-ray Diffraction (XRD) analysis, as illustrated in Figure 1. The XRD pattern confirms that the nanoparticles exhibit a single-phase structure, specifically Zn_2_SnO_4_, with no evidence of secondary phases such as SnO_2_, ZnO, or ZnSnO_3_. All the observed diffraction peaks align with the Fd-3m cubic-spinel structure typical of Zn_2_SnO_4_. Notable high-intensity diffraction peaks are observed at approximately 17.775°, 29.17°, 35.177°, 41.682°, and 60.505°. According to the reference data from the PDF and ICDD (International Centre for Diffraction Data) sheets, these peaks correspond to the (111), (311), (222), (400), and (440) planes, all characteristic of a cubic crystal system. The average crystallite size of the synthesized Zn_2_SnO_4_ nanoparticles was determined using the Debye–Scherrer equation (Equation (1)), yielding an estimated size of approximately 191.508 Å:

(1) $$D=\frac{K\times \lambda }{\beta \times \cos\theta }$$

where 

D = average grain size (nm);

K = Scherrer constant, which denotes the shape of the particle and has the value of 0.9;

λ = X-ray wavelength (1.54184 Å);

β = full-width at half-maximum of the observed peaks (FWHM);

θ = diffraction angle (°).

Table 1 presents the average crystallite size values obtained for Zn_2_SnO_4_, Zn_2_SnO_4_@SiO_2_, and Zn_2_SnO_4_@SiO_2_@5-FU nanoparticles.

Figure 1 presents the X-ray diffraction patterns for the synthesized samples. In the Zn_2_SnO_4_@SiO_2_ and Zn_2_SnO_4_@SiO_2_@5-FU samples, a slight shift in the diffraction peaks is noticeable, which can be attributed to the SiO_2_ coating on the particles. Additionally, the peak intensities exhibit a moderate reduction compared to the pure Zn_2_SnO_4_ sample, indicating a decrease in crystallinity for the Zn_2_SnO_4_@SiO_2_ and Zn_2_SnO_4_@SiO_2_@5-FU samples. This observation confirms that the Zn_2_SnO_4_ nanoparticles were successfully encapsulated with SiO_2_ and subsequently with 5-FU. The XRD pattern of Zn_2_SnO_4_@SiO_2_@5-FU presents an intense peak at 28.603°, characteristic of the crystalline nature of 5-FU.

### 2.2. Scanning Electron Microscopy (SEM)

**Figure 2:**
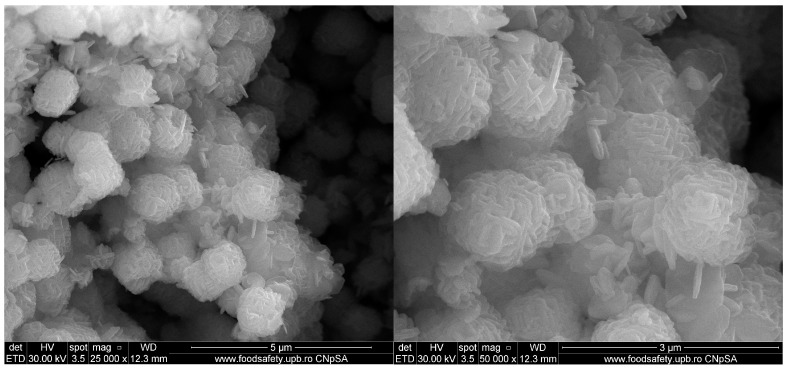
SEM micrographs of Zn_2_SnO_4_.

**Figure 3:**
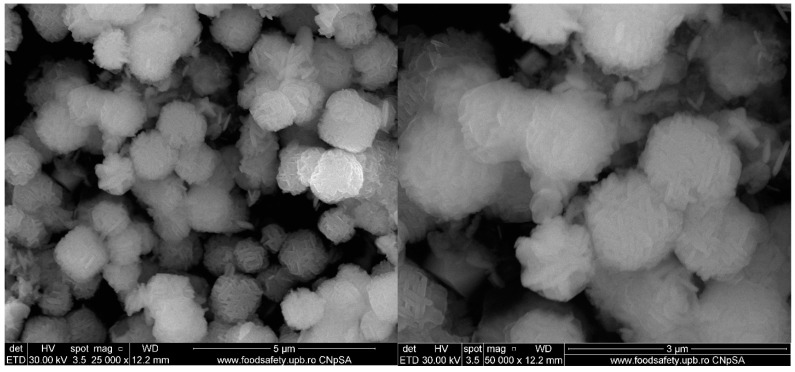
SEM micrographs of Zn_2_SnO_4_@SiO_2_.

**Figure 4:**
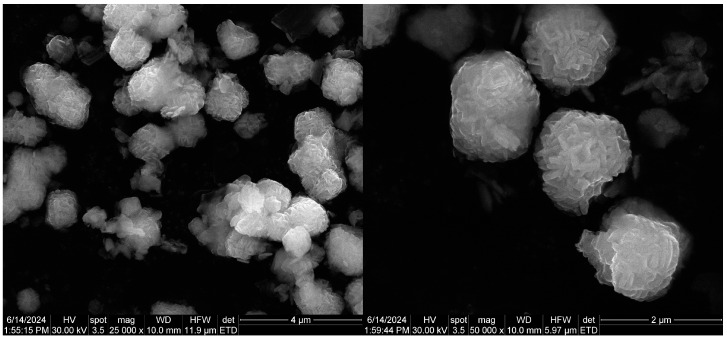
SEM micrographs of Zn_2_SnO_4_@SiO_2_@5-FU.

**Figure 5:**
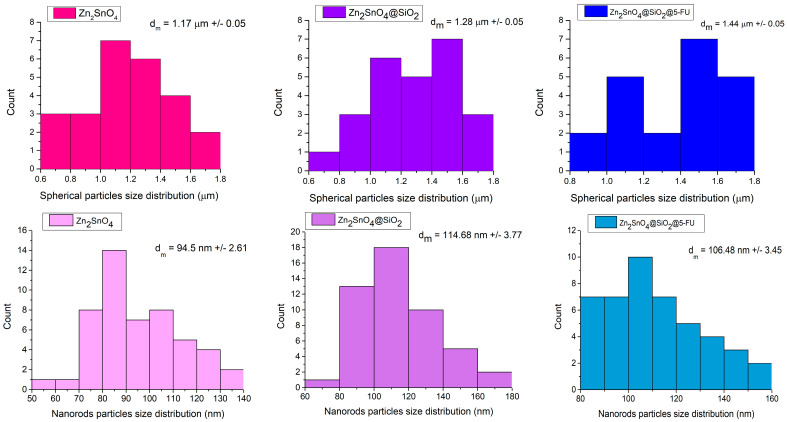
Particle size distribution of spheres (µm) and nanorods (nm), as computed from the corresponding SEM micrograph of each material. Size measurements were performed using ImageJ software V 1.53, analyzing multiple images to ensure accuracy and reproducibility.

SEM micrographs reveal that the Zn_2_SnO_4_ particles exhibit a spherical morphology formed by the aggregation of nanorods, as depicted in Figure 2 at magnifications of 25,000× and 50,000×. These particles demonstrate a tendency to agglomerate and display dimensional uniformity, suggesting a high level of homogeneity in shape. Dimensional analysis was conducted for both spherical and rod-like morphologies, with average particle sizes of 1.17, 1.28, and 1.44 µm for spherical particles and 94.5, 114.68, and 106.48 nm for the nanorods. Following the SiO_2_ coating, a shell layer becomes visible on the surface of the particles, as shown in the micrographs in Figure 3. With the subsequent incorporation of 5-FU, the coating layer appears even more defined, as indicated in Figure 4. This suggests that 5-FU interacts with the silica shell, potentially forming a denser and more uniform layer around the particles. Notably, adding the silica shell and 5-FU does not significantly impact the size or morphology of the original Zn_2_SnO_4_ particles, indicating that the coating and drug incorporation processes preserve the physical dimensions and shape. Figure 5 illustrates the size distribution for both spherical particles and the nanorod-aggregated spheres.

### 2.3. Energy Dispersive Spectroscopy (EDS)

**Figure 6:**
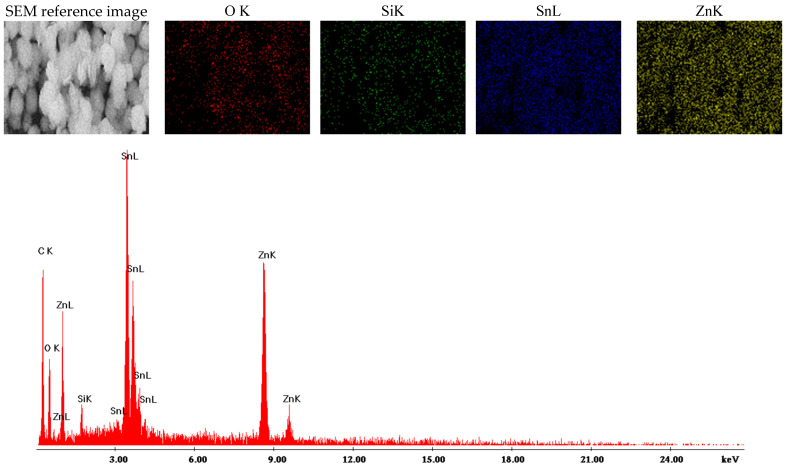
Elemental mapping obtained for Zn_2_SnO_4_@SiO_2_.

**Figure 7:**
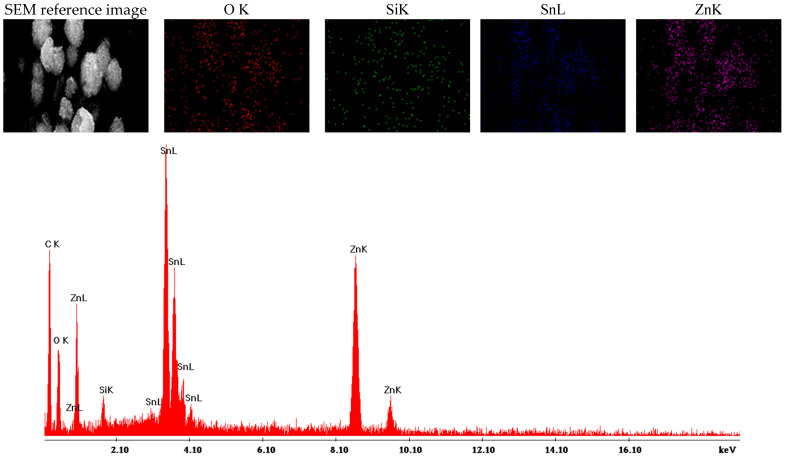
Elemental mapping obtained for Zn_2_SnO_4_@SiO_2_@5-FU.

SEM characterization is frequently complemented by energy-dispersive X-ray spectroscopy (EDS), which includes elemental mapping to visually distinguish the presence of each element within the samples. For Zn_2_SnO_4_@SiO_2_, the elemental mapping reveals the presence of O, Si, Sn, and Zn, with each element represented by distinct colors, confirming the sample’s composition. The EDS spectra, illustrated in Figure 6, corroborate these findings by displaying characteristic peaks for each detected element. The mapping results indicate a uniform distribution of all elements across the sample. The Zn_2_SnO_4_@SiO_2_@5-FU sample underwent similar analysis, yielding comparable results regarding elemental composition. EDS mapping identified O, Si, Sn, and Zn, each highlighted in distinct colors in Figure 7. Additionally, the EDS spectra confirm that the inclusion of 5-FU does not alter the elemental composition, maintaining consistent characteristics as observed in the Zn_2_SnO_4_@SiO_2_ sample. Additionally, the EDS spectra confirm that the inclusion of 5-FU does not alter the elemental composition, maintaining consistent characteristics with the Zn_2_SnO_4_@SiO_2_ sample. This observation, combined with FTIR analysis, suggests that 5-FU is primarily physically adsorbed onto the Zn_2_SnO_4_@SiO_2_ surface without forming new chemical bonds.

### 2.4. Fourier Transform Infrared Spectroscopy (FTIR)

**Figure 8:**
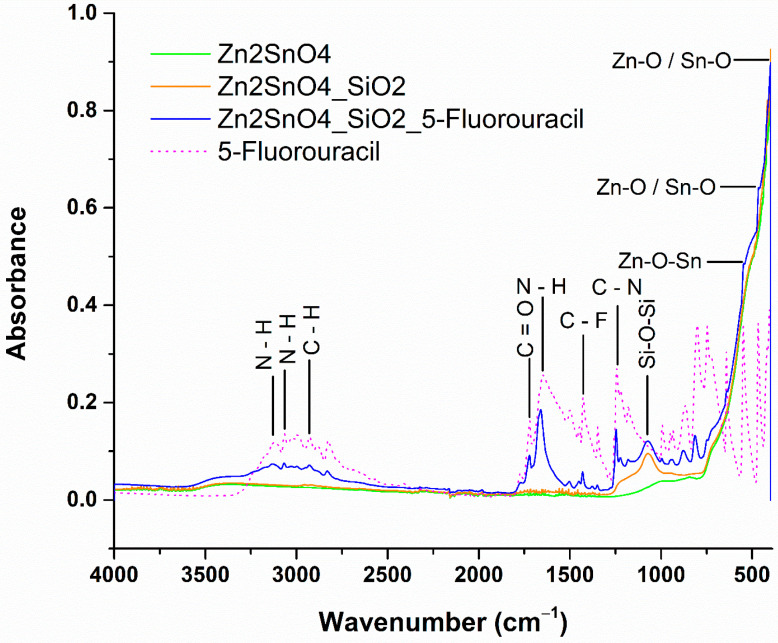
FTIR spectra results for Zn_2_SnO_4_, Zn_2_SnO_4_@SiO_2_, Zn_2_SnO_4_@SiO_2_@5-FU, and 5-FU substance.

Valuable insights into the vibrational characteristics of atomic bonds within the material can be gained through Fourier Transform Infrared (FTIR) analysis. Measurements for all powder samples were conducted over a wavenumber range of 400 to 4000 cm^−1^. The resulting FTIR spectra, which provide information on the functional groups and bonding present in the samples, are illustrated in Figure 8.

The FTIR analysis of the zinc stannate sample reveals a prominent vibrational band at 590 cm^−1^, which corresponds to the symmetric stretching vibration of ZnO and SnO_2_ groups, indicating the Zn–O–Sn bonding characteristic of Zn_2_SnO_4_. This absorption band strongly supports the successful formation of the zinc stannate compound, which is consistent with its expected properties. Additionally, wavenumbers at 463 cm^−1^ and 400 cm^−1^ are attributed to Zn–O and Sn–O chemical groups, respectively, confirming the synthesis of zinc stannate. These vibrational bands are consistently observed in all the synthesized samples, indicating that zinc stannate is the primary constituent in the formulations [36,37]. For Zn_2_SnO_4_@SiO_2_ and Zn_2_SnO_4_@SiO_2_@5-FU samples, a distinct vibrational band at 1072 cm^−1^ is observed, corresponding to the Si–O–Si functional group, which confirms the presence of the SiO_2_ coating layer. The incorporation of 5-FU in the Zn_2_SnO_4_@SiO_2_@5-FU structure is validated by several absorption bands in the FTIR spectra, consistent with the reference spectrum for 5-FU. Notably, N–H bending vibrations are detected at 3130 and 3067 cm^−1^, along with a similar bending band at 1600 cm^−1^, characteristic of the 5-FU compound. Additionally, C–H stretching appears at 2929 cm^−1^, while the C=O functional group is identified at 1772 cm^−1^. Strong evidence for 5-FU integration is further provided by the stretching vibration at 1430 cm^−1^, attributed to the fluoro compound C–F, and a band at 1240 cm^−1^ associated with the C–N functional group [38,39].

### 2.5. Dynamic Light Scattering (DLS)

Zeta potential measurements highlight that Zn_2_SnO_4_ nanoparticles possess a negatively charged surface, with an average value of −17.93 mV, indicating superior stability in solution. The negative surface charge not only ensures dispersion stability but also enhances their potential for interacting with biological membranes, including both bacterial and cancer cells. These interactions are key to the nanoparticles’ antimicrobial and cytotoxic effects. The addition of a SiO_2_ layer enhances the stability of the samples in solution, forming a protective shell around the Zn_2_SnO_4_ nanoparticles. This is reflected in a zeta potential measurement of approximately −21.45 mV for the Zn_2_SnO_4_@SiO_2_ sample. The silica coating helps to prevent nanoparticle aggregation and sedimentation, ensuring even dispersion within the solution.

**Figure 9:**
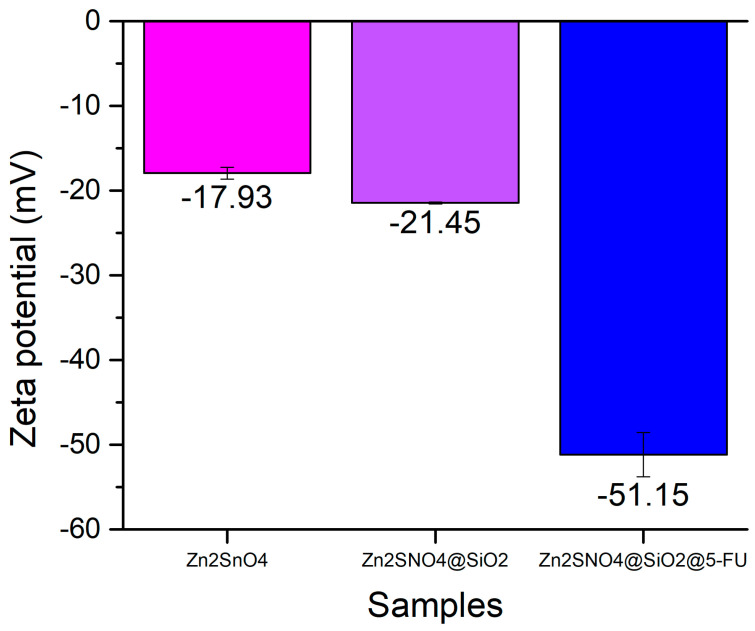
Zeta potential (mV) results of Zn_2_SnO_4_, Zn_2_SnO_4_@SiO_2_, and Zn_2_SnO_4_@SiO_2_@5-FU samples.

Furthermore, incorporating 5-FU onto the nanoparticles’ surface further increases stability, resulting in a zeta potential of −51.15 mV. This significant increase in stability is attributed to the interaction between 5-FU and the silica shell, leading to a highly stable solution, as depicted in Figure 9**.** The enhanced negative zeta potential also correlates with the observed antimicrobial and antitumor effects as the negatively charged nanoparticles strongly interact with positively charged regions of bacterial and cancer cell membranes. This facilitates membrane disruption, increased oxidative stress, and improved drug delivery efficiency, leading to heightened antibacterial and anticancer activity. Such enhanced stability is useful for medical applications, ensuring that the nanoparticles remain structurally intact and functionally effective until they reach their target site within the body.

The hydrodynamic diameter measurements confirm that the particle size increased following the addition of both SiO_2_ and 5-FU, indicating successful coverage with the silica shell and the subsequent incorporation of the antitumoral and antibacterial agent. For the uncoated Zn_2_SnO_4_ nanoparticles, the hydrodynamic diameter was estimated to be 1636.83 nm. Upon coating with SiO_2_, the hydrodynamic diameter increased to approximately 1839.06 nm, demonstrating the successful formation of the silica layer around the Zn_2_SnO_4_ nanoparticles.

**Figure 10:**
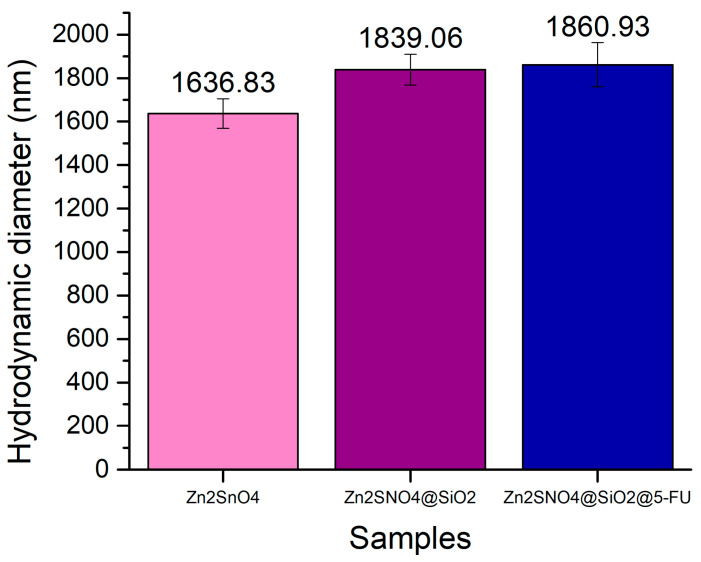
Hydrodynamic diameter (nm) results of Zn_2_SnO_4_, Zn_2_SnO_4_@SiO_2_, and Zn_2_SnO_4_@SiO_2_@5-FU samples.

Furthermore, after the integration of 5-FU, the dynamic light scattering (DLS) results showed similar hydrodynamic diameter characteristics to those of the Zn_2_SnO_4_@SiO_2_ sample, suggesting that 5-FU was uniformly incorporated into the SiO_2_ surface (Figure 10). 

### 2.6. Antimicrobial Assay

**Figure 11:**
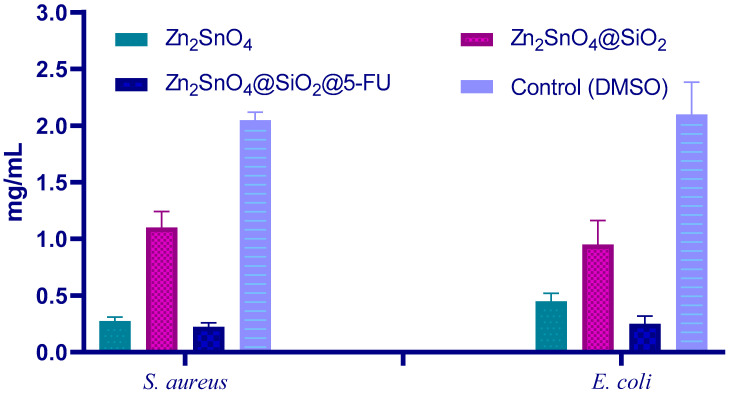
The minimum inhibitory concentration (MIC) of Zn_2_SnO_4_, Zn_2_SnO_4_@SiO_2_, and Zn_2_SnO_4_@SiO_2_@5-FU samples against *S. aureus* and *E. coli*.

The MIC values presented in Figure 11 reveal the antibacterial performance of the Zn_2_SnO_4_-based nanoparticles against *S. aureus* (Gram-positive) and *E. coli* (Gram-negative). Against both *S. aureus* and *E. coli*, the Zn_2_SnO_4_@SiO_2_@5-FU formulation demonstrates the lowest MIC, indicating the highest antibacterial efficacy among the tested samples. This result highlights the contribution of 5-FU functionalization, which enhances the antimicrobial activity through its chemotherapeutic and antibacterial properties. However, the Zn_2_SnO_4_@SiO_2_ formulation shows a slightly reduced antibacterial activity compared to Zn_2_SnO_4_, likely due to the silica shell partially shielding the core and reducing the availability of active species such as ROS and Zn^2+^/Sn^4+^ ions, which are essential for antimicrobial efficacy.

The MIC results for *E. coli* are comparable to those observed for *S. aureus*, suggesting that the formulations are equally effective against both bacterial types despite the structural differences between Gram-positive and Gram-negative bacteria. The enhanced efficacy of Zn_2_SnO_4_@SiO_2_@5-FU across both strains underscores the potential of this multifunctional nanoparticle system for broad-spectrum antibacterial applications. The DMSO control demonstrates significantly higher MIC values, confirming that the observed antibacterial effects are attributable to the nanoparticle formulations. These findings suggest that Zn_2_SnO_4_@SiO_2_@5-FU is a promising antibacterial agent with efficacy against both Gram-positive and Gram-negative bacteria.

The antimicrobial mechanism of Zn_2_SnO_4_@SiO_2_@5-FU nanoparticles against Gram-positive and Gram-negative bacteria can be attributed to their distinct structural and functional features. For Gram-positive bacteria such as *S. aureus*, the thick and porous peptidoglycan layer facilitates the penetration of nanoparticles and their active species. Reactive oxygen species generated by Zn_2_SnO_4_ nanoparticles interact with bacterial cell walls, proteins, and DNA, inducing oxidative stress, which disrupts vital cellular processes [20,24]. Additionally, the release of Zn^2+^ and Sn^4+^ ions interferes with bacterial enzymatic activities by binding to sulfhydryl (-SH) groups in membrane proteins, destabilizing the membrane and impairing metabolic pathways [23,28]. These processes, combined with the antimicrobial properties of 5-FU, enhance membrane disruption and lead to bacterial lysis, contributing to the observed efficacy against Gram-positive strains [40].

In Gram-negative bacteria like *E. coli*, the presence of an outer membrane composed of lipopolysaccharides serves as a protective barrier, making these bacteria inherently more resistant to antimicrobial agents [41]. However, the Zn_2_SnO_4_@SiO_2_@5-FU nanoparticles overcome this barrier through multiple mechanisms. ROS produced by the nanoparticles penetrate the outer membrane and cause oxidative damage [20,24], while Zn^2+^ and Sn^4+^ ions interact with negatively charged LPS molecules, destabilizing the outer membrane and increasing permeability [28,41]. This enables the nanoparticles and 5-FU to access the thinner peptidoglycan layer and the underlying cellular components. The incorporation of 5-FU further disrupts bacterial DNA synthesis and metabolic pathways, amplifying the overall antimicrobial effect [40,42]. These synergistic mechanisms allow Zn_2_SnO_4_@SiO_2_@5-FU nanoparticles to achieve comparable efficacy against both Gram-positive and Gram-negative bacteria, showcasing their potential as broad-spectrum antibacterial agents [42].

### 2.7. Cytotoxicity Assay

To evaluate the cytotoxicity activity of Zn_2_SnO_4_, Zn_2_SnO_4_@SiO_2_, and Zn_2_SnO_4_@SiO_2_@5-FU powders on the A-431 human epidermoid carcinoma cell line, cells were treated for 24 h with varying concentrations of these nanoparticles. Post-treatment cell metabolic activity was assessed using the MTT spectrophotometric assay to determine the cytotoxicity of each powder on A-431 tumor cells.

**Figure 12:**
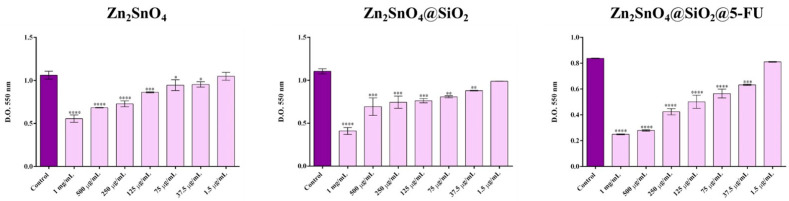
Graphics representing the viability of A-431 tumor cells after 24 h post-treatment with different concentrations of Zn_2_SnO_4_, Zn_2_SnO_4_@SiO_2_, and Zn_2_SnO_4_@SiO_2_@5-FU powders (**** *p* ≤ 0.0001 control vs. sample, *** *p* ≤ 0.001 control vs. sample, ** *p* ≤ 0.01 control vs. sample, and * *p* ≤ 0.05 control vs. sample).

The cytotoxicity assessment (Figure 12) revealed that all Zn_2_SnO_4_-based powders exerted a toxic effect on A-431 tumor cells, with the cytotoxicity increasing in a dose-dependent manner. While all three powders significantly reduced cell viability at both high and low concentrations, a marked decrease in cell viability was observed at concentrations as low as 37.5 µg/mL. At a 1.5 µg/mL concentration, no significant cytotoxic effect was detected compared to the untreated control across all samples. Despite similarities in their cytotoxic profiles, Zn_2_SnO_4_@SiO_2_@5-FU exhibited the strongest cytotoxicity, causing a more pronounced decrease in cell viability than Zn_2_SnO_4_ and Zn_2_SnO_4_@SiO_2_ at equivalent concentrations. At the highest concentration tested (1 mg/mL), Zn_2_SnO_4_ reduced cell viability by a factor of 1.9 compared to the control, Zn_2_SnO_4_@SiO_2_ by 2.75 times, and Zn_2_SnO_4_@SiO_2_@5-FU by 3.45 times, with significant cytotoxicity also observed at lower treatment concentrations.

Furthermore, the cytotoxicity data were utilized to estimate the lethal dose 50 (LD_50_)—the concentration required to kill 50% of the cells exposed. The LD_50_ for Zn_2_SnO_4_ was 1 mg/mL, while the LD_50_ for Zn_2_SnO_4_@SiO_2_ could not be precisely determined but fell between 500 µg/mL and 1 mg/mL. In contrast, the LD_50_ for Zn_2_SnO_4_@SiO_2_@5-FU was 250 µg/mL, indicating a significantly higher cytotoxic potential for the 5-FU-incorporated sample due to the lower LD_50_ value.

**Figure 13:**
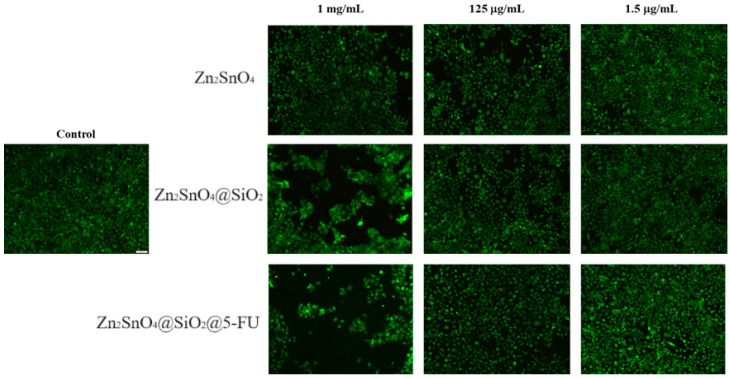
Fluorescence microscopy images showing living (green) and dead (red) A-431 tumor cells after 24 h post-treatment with different concentrations of Zn_2_SnO_4_, Zn_2_SnO_4_@SiO_2_, and Zn_2_SnO_4_@SiO_2_@5-FU powders (10× magnification).

Fluorescence microscopy, using the Live/Dead staining of A-431 tumor cells, confirmed the MTT assay findings (Figure 13). The Zn_2_SnO_4_ powder displayed mild cytotoxic effects compared to the control. In the Zn_2_SnO_4_@SiO_2_ treatment, a noticeable decline in cell viability was observed, with cells losing their characteristic compact cluster formation, which is typical for tumor cell organization. The Zn_2_SnO_4_@SiO_2_@5-FU powder, particularly at higher concentrations (1 mg/mL), showed the most substantial cytotoxic impact, with significantly fewer viable cells, which were organized into small, dispersed clusters, in stark contrast to the untreated control sample.

## 3. Discussion

Traditional bone fillers commonly used in dental maxillofacial reconstruction often include materials such as calcium phosphate cement, hydroxyapatite, and bioactive glass [43,44]. While these materials provide basic support for bone regeneration, they frequently exhibit limitations such as brittleness, fragility, a high rate of deterioration that impacts cell growth, susceptibility to infection, poor integration with host bone, and limited antimicrobial or antitumor capabilities. Structural and biocompatibility concerns, particularly the lack of effective barriers against microbial contamination and inadequate mechanical stability in the implanted bone fracture, remain major drawbacks in conventional fillers [45]. To increase the limited biodegradability and antibacterial action of the bone fillers, metal oxides such as MgO, ZnO, and ZrO_2_ have been used [46,47,48,49,50]. The synthesized Zn_2_SnO_4_@SiO_2_@5-FU nanoparticles demonstrate clear advantages over traditional fillers regarding structural stability and biocompatibility. The presence of a SiO_2_ coating significantly improves stability and dispersibility, addressing common issues of aggregation seen in conventional materials. SiO_2_ is an inorganic compound suitable for coating over inner core materials because of its superior thermal stability, capacity to tolerate high temperatures, and protection against core deterioration in an acidic environment. S. Lims et al. used SiO_2_ as a protective shell over Zn_2_SnO_4_ nanoparticles, modifying their functionality and reactivity, which improves the stability of the inner core and prevents the aggregation of core particles. The Stöber method employed to cover the synthesized Zn_2_SnO_4_ nanoparticles has several significant benefits, including high purity, environmental friendliness, and the simplicity of controlling shape and size [51,52].

Furthermore, Zn_2_SnO_4_@SiO_2_@5-FU nanoparticles offer enhanced integration potential due to their high biocompatibility and targeted functionalities, which are not found in standard bone fillers. Infection remains a critical concern in dental reconstructions using traditional fillers, which often lack intrinsic antibacterial properties. In contrast, Zn_2_SnO_4_@SiO_2_@5-FU nanoparticles display superior antibacterial activity, particularly against Gram-positive bacteria such as *S. aureus*. Y. Lakshmi et al. [28] reported the antibacterial activity of Zn2SnO4 nanoparticles against Gram-positive bacteria (*B. subtilis* and *S. aureus*) and Gram-negative bacteria (*P. aeruginosa* and *E. coli*). The results indicate a significant antibacterial effect against both strains at higher concentrations (10 mg/L) [17].

The incorporation of 5-FU enhances this effect, offering a significant advantage in reducing post-surgical infection risks. The interaction between the SiO_2_ and 5-FU was analyzed in numerous studies. C. Ding et al. presented that the dispersion of SiO_2_ nanoparticles in hydrogels can increase the resistance of 5-FU release into the surrounding liquid and considerably lower the burst release [53]. In this context, Zn_2_SnO_4_@SiO_2_@5-FU represents a more reliable choice for maintaining sterile conditions in clinical applications. Biofilm formation is a major limitation of conventional bone fillers, often leading to persistent infections and compromised surgical outcomes. 5-FU exhibits antibacterial properties against *Streptococcus suis*, *Staphylococcus aureus*, and *E. coli*. In *Pseudomonas aeruginosa* and *E. coli*, it can lessen bacterial pathogenicity and prevent the growth of biofilms [40,42,54]. In this study, the Zn_2_SnO_4_@SiO_2_@5-FU nanoparticles showed notable efficacy in inhibiting biofilm formation, a feature that directly addresses the shortcomings of standard fillers. This capability is crucial for long-term clinical success as it ensures that the material maintains a sterile environment, preventing bacterial colonization and related complications. Unlike conventional bone fillers, Zn_2_SnO_4_@SiO_2_@5-FU nanoparticles possess distinct antitumor properties due to the inclusion of 5-FU, a chemotherapeutic agent. This dual functionality enables the filler to contribute to bone regeneration and the local treatment of cancerous tissues, which is particularly relevant in oral cancer contexts. A study showed that 5-FU can increase programmed death-ligand 1 (PD-L1) expression in a cell model of oral squamous cell carcinoma, influencing treatment response and aiding chemoresistance [55]. In another research study, SiO_2_ nanoparticles functionalized with chitosan and PEG and loaded with 5-FU demonstrated favorable uptake in cancer cell lines. Forty-eight-hour exposure treatments resulted in strong, induced cytotoxic, apoptotic, and cell-cycle distribution shift events in colon, breast, and cervical cancer cells that experienced rapid apoptotic events and cell-cycle distribution shifts. Furthermore, at therapeutically relevant dosages (0.15–0.18 mg_5-FU_/mg_SiO2_), cytotoxicity studies demonstrated an effective reduction in the malignant cell population [56]. The ability to combine bone healing with targeted tumor therapy offers a unique advantage in complex dental reconstructions involving oncological concerns. The Zn_2_SnO_4_@SiO_2_@5-FU nanoparticles exhibit superior mechanical stability under the conditions expected in dental applications. This resilience surpasses that of many conventional fillers, which often suffer from structural weaknesses [41]. The enhanced mechanical properties of Zn_2_SnO_4_@SiO_2_@5-FU contribute to better bone regeneration and integration, leading to more robust and lasting outcomes in maxillofacial reconstructions. By promoting mineral formation at the bonded dentin/restoration interface and lessening the effects of acid-producing bacteria, these nanoparticles can increase dental restorations’ strength and fatigue resistance [57]. Despite the advantages, potential challenges remain in the synthesis and use of Zn_2_SnO_4_@SiO_2_@5-FU. The functionalization processes employed in order to be environmentally compatible [58] are more complex than those required for standard fillers, which could impact scalability and clinical adoption. However, the enhanced therapeutic benefits justify these complexities, and further refinement could optimize the material for broader clinical use. Future studies should also consider potential cytotoxic effects at varying doses to ensure safety. Looking forward, in vivo studies will be critical to validate the performance of Zn_2_SnO_4_@SiO_2_@5-FU nanoparticles in real clinical scenarios. Additionally, combining these nanoparticles with other bioactive materials could amplify their efficacy, particularly in complex reconstructions. These developments can potentially revolutionize the field of dental and maxillofacial surgery, offering a more comprehensive solution to bone regeneration and infection prevention in reconstructive applications.

## 4. Materials and Methods

### 4.1. Materials

The materials used for the synthesis of Zn_2_SnO_4_ nanoparticles included zinc nitrate (Zn(NO_3_)_2_·6H_2_O), tin tetrachloride (SnCl_4_), sodium hydroxide (NaOH), and distilled water. The SiO_2_ shell was formed using ammonia (NH_3_) solution and tetraethyl orthosilicate (TEOS). Surface modification of the SiO_2_ shell was achieved using 5-Fluorouracil (5-FU), which was attached through hydrogen bonding and Van der Waals interactions. All chemicals were sourced from Sigma-Aldrich (Merck Group, Darmstadt, Germany).

### 4.2. Synthesis of Zn_2_SnO_4_ Nanoparticles

To synthesize Zn_2_SnO_4_ nanoparticles, 2.9749 g of zinc nitrate (Zn(NO_3_)_2_·6H_2_O) and 0.7 mL of tin tetrachloride (SnCl_4_) were employed as sources of zinc and tin, respectively. Each chemical was dissolved separately in 50 mL of distilled water to form two clear solutions. These solutions were then combined and mixed at 300 rpm for 2 h. A solution of 4 g of sodium hydroxide (NaOH) dissolved in 50 mL of distilled water was added dropwise to the Zn-Sn mixture while undergoing magnetic stirring. The mixture was maintained under stirring for an additional 2 h at 200 rpm before being transferred to SynthWAVE equipment for further processing. The synthesized composite powder was washed three times with ethanol and distilled water, followed by centrifugation at 6000 rpm/~3000× *g* for 5 min to eliminate any residuals. The precipitate was then calcined at 100 °C for 7 h.

### 4.3. Synthesis of Zn_2_SnO_4_@SiO_2_ Nanoparticles

To create a SiO_2_ shell around the zinc stannate particles, the Stöber method was employed using the pre-synthesized Zn_2_SnO_4_ powder. A quantity of 0.285 g of Zn_2_SnO_4_ nanoparticles was mixed with 10 mL of ethanol and 10 mL of distilled water, and the mixture was subjected to magnetic stirring for 10 min. Simultaneously, 1 mL of aqueous ammonia solution was prepared and added dropwise to the nanoparticle suspension. Following this, 1.5 mL of tetraethyl orthosilicate (TEOS) was introduced into the mixture, which was then left under continuous magnetic stirring for 36 h. The resulting compound was washed three times with ethanol and distilled water, with each cycle followed by centrifugation at 6000 rpm/~3000× *g* for 5 min. The final precipitate was heated in an oven at 140 °C for 10 h to complete the process.

### 4.4. Synthesis of Zn_2_SnO_4_@SiO_2_@5-FU Nanoparticles

The SiO_2_-coated Zn_2_SnO_4_ nanoparticles were subsequently functionalized with 5-Fluorouracil (5-FU) using hydrogen and Van der Waals interactions. To achieve this, 5-FU was dissolved in ethanol and subjected to ultrasonic treatment at 80 °C, for 10 min, at 100 W. The 5-FU was added in a 1% mass ratio relative to the Zn_2_SnO_4_@SiO_2_ nanoparticles. The mixture was then manually ground with a mortar and pestle until the ethanol had completely evaporated. This surface modification ensured the controlled and stable attachment of 5-FU molecules to the SiO_2_-coated Zn_2_SnO_4_ surface. The modified nanoparticles demonstrated enhanced biocompatibility, greater bioavailability, and increased efficacy in targeting cancer cells.

### 4.5. Characterization Methods

#### 4.5.1. X-Ray Diffraction (XRD)

The crystallinity and crystal parameters investigation of the Zn_2_SnO_4_, Zn_2_SnO_4_@SiO_2_, and Zn_2_SnO_4_@SiO_2_@5-FU was performed through an X-ray diffraction technique, using in this sense a PANalytical Empyrean model diffractometer purchased from PANalytical, Almelo, the Netherlands, equipped with a hybrid monochromator (2xGe 220) on the incident side and parallel plate collimator mounted on PIXcel 3D detector on the diffracted side. Grazing Incidence X-ray Diffraction (GIXRD) measurements were performed at room temperature, with an angle of incidence ω = 0.5° for Bragg angle values of 2θ between 10° and 80°, using Cu Kα radiation with λ = 1.5406 Å (40 mA and 45 kV).

#### 4.5.2. Scanning Electron Microscopy (SEM)

To examine the morphological characteristics of Zn_2_SnO_4_, Zn_2_SnO_4_@SiO_2_, and Zn_2_SnO_4_@SiO_2_@5-FU nanoparticles, a Scanning Electron Microscopy (SEM) analysis was conducted. The samples were mounted on carbon-coated slides and placed in the analysis chamber of an Inspect F50 scanning electron microscope, acquired from Thermo Fisher—FEI (Eindhoven, The Netherlands). The images were obtained by capturing the secondary electron emission and electron beam scattering, using an accelerating energy of 30 keV.

#### 4.5.3. Dynamic Light Scattering (DLS)

Dynamic Light Scattering (DLS) measurements were conducted using a DelsaMax Pro device (Backman Coulter, Brea, CA, USA), equipped with a 532 nm laser. The nanoparticle powders were dispersed in ultrapure water at room temperature. To ensure optimal dispersion, all samples underwent ultrasonic treatment for 10 min in an ultrasonic bath prior to measurement.

#### 4.5.4. Fourier Transform Infrared Spectroscopy (FTIR)

Fourier Transform Infrared (FTIR) analysis was conducted to identify the functional compositional groups present in the samples. A Thermo iN10-MX FTIR spectrometer (Thermo Fischer Scientific, Waltham, MA, USA), equipped with a ZnSe crystal, was used for the measurements. The spectra were collected over a range of 4000 to 400 cm^−1^. The instrument was sourced from Thermo Fisher Scientific, Waltham, MA, USA.

#### 4.5.5. Antimicrobial Assay

The minimum inhibitory concentration (MIC) was determined using the microdilution method. This procedure was conducted in 96-well plates using Trypticase Soy Broth (TSB) liquid medium or a simple broth, with a final volume of 150 µL per well. Serial binary dilutions were prepared from each test solution, with concentrations ranging from 2 mg/mL to 0.0078 mg/mL (i.e., 2, 1, 0.5, 0.25, 0.125, 0.0625, 0.0156, and 0.0078 mg/mL). After preparing the appropriate dilutions, 15 µL of a microbial suspension with a standard density (0.5 McFarland for bacteria, 1.0 McFarland for yeasts) was added to each well. The same dilution series was prepared for the solvent used to create the test dilutions, such as DMSO.

The 96-well plates were incubated at 37 °C for 24 h in a humid chamber. Results were assessed through macroscopic observation and/or by spectrophotometric measurement at 600 nm. For spectrophotometric analysis, 100 µL of each sample was transferred to a new 96-well plate for accurate readings.

#### 4.5.6. In Vitro Cytotoxicity

The antitumor potential of Zn_2_SnO_4_, Zn_2_SnO_4_@SiO_2_, and Zn_2_SnO_4_@SiO_2_@5-FU powders was assessed using the A-431 human epidermoid carcinoma cell line. Cells were maintained in Dulbecco’s Modified Eagle’s Medium (DMEM), supplemented with 10% fetal bovine serum (FBS) and 1% antibiotic-antifungal mixture, under standard conditions of 37 °C and 5% CO_2_.

For treatment application, the cells were detached from the culture surface using enzymatic-chemical trypsin/EDTA detachment, counted using a hemocytometer, and seeded at a density of 1 × 10^4^ cells per well in sterile 96-well plates. The cells were incubated for 24 h under standard conditions before applying treatments. A 2 mg/mL stock solution was prepared in a complete culture medium for each of the three tested powders and then sterilized by passing through a 0.22 µM syringe filter. This stock solution was used to prepare treatment solutions with concentrations of 1 mg/mL, 500 µg/mL, 250 µg/mL, 125 µg/mL, 75 µg/mL, 37.5 µg/mL and 1.5 µg/mL. The culture medium was removed from the wells and replaced with the treatment solutions, while the fresh culture medium was used as a control. After 24 h of treatment, two key assessments were conducted.

The MTT assay was used to quantify cell viability. This colorimetric assay measures cell viability and proliferation based on the ability of metabolically active cells to reduce yellow tetrazolium salt (MTT) into purple formazan crystals, a process that occurs at the mitochondrial level via NADH-dependent oxidoreductases. After 24 h of treatment, the culture medium was aspirated from the cell monolayers and replaced with a freshly prepared MTT solution, dissolved in a serum-free medium to a final concentration of 1 mg/mL. The plates were incubated for 4 h under standard conditions. The MTT solution was then removed, and the resulting formazan crystals were dissolved in isopropanol. The optical density (OD) of the solutions was measured at a wavelength of 550 nm using a FlexStation III multimode plate reader (Molecular Devices, San Jose, CA, USA). Statistical analysis was conducted with GraphPad Prism V9 software using an ANOVA test with Bonferroni correction. Data were presented as the mean of three biological replicates ± standard deviation, with a statistical significance threshold of *p* < 0.05.

The Live/Dead assay was used to investigate cell viability and cellular organization under the influence of the treatments. This qualitative method allows for the simultaneous identification of live and dead cells using calcein and ethidium bromide (EtBr). The assay assesses intracellular esterase activity and plasma membrane integrity. Calcein AM, a nonfluorescent compound, penetrates viable cell membranes and is converted by intracellular esterases into calcein, which fluoresces green. EtBr only enters cells with damaged membranes and binds to nucleic acids, emitting a red fluorescence. The Live/Dead Viability Cytotoxicity Kit for mammalian cells (Invitrogen, Thermo Fischer Scientific, Waltham, MA, USA) was employed, with the staining solution prepared by diluting the kit components in serum-free medium to a final concentration of 2 µM calcein AM and 4 µM EtBr. After 15 min of incubation at room temperature in the dark, the samples were examined under an Olympus IX73 inverted fluorescence microscope. Images were captured and processed using CellSense Imaging Software V 8.0.2 (Olympus, Tokyo, Japan).

## 5. Conclusions

This study highlights that Zn_2_SnO_4_@SiO_2_@5-FU nanoparticles are a promising multifunctional additive for bone fillers in dental maxillofacial reconstruction. The synthesis process effectively retained the crystallinity of Zn_2_SnO_4_ even after SiO_2_ coating and 5-FU functionalization, as confirmed by X-ray diffraction analysis. The SiO_2_ layer significantly enhanced the stability of the nanoparticles in aqueous environments, while 5-FU improved biocompatibility and exhibited effective antimicrobial and antitumor properties. Antibacterial tests indicated strong inhibitory activity against Gram-positive bacteria, making these nanoparticles suitable for infection prevention in clinical settings. Additionally, cytotoxicity studies on the A-431 human epidermoid carcinoma cell line demonstrated a dose-dependent reduction in cell viability, highlighting the potential of these nanoparticles for targeted cancer therapy. The successful inhibition of biofilm formation further supports the utility of Zn_2_SnO_4_@SiO_2_@5-FU in preventing bacterial colonization. These results suggest that Zn_2_SnO_4_@SiO_2_@5-FU nanoparticles could serve as a valuable component in bone fillers, enhancing their therapeutic and antimicrobial performance in dental and maxillofacial reconstruction applications. Future research should explore in vivo efficacy to confirm these findings and optimize clinical outcomes.

## Figures and Tables

**Table 1:** The average crystallite size of Zn_2_SnO_4_-based nanoparticles.

Sample	Zn_2_SnO_4_	Zn_2_SnO_4_@SiO_2_	Zn_2_SnO_4_@SiO_2_@5-FU
Average crystallite size (Å)	191.508	217.575	209.065

## Data Availability

The original contributions presented in this study are included in the article. Further inquiries can be directed to the corresponding author.
